# Why Victimized Employees Become Less Engaged at Work: An Integrated Model for Testing the Mediating Role of Sleep Quality

**DOI:** 10.3390/ijerph18168468

**Published:** 2021-08-11

**Authors:** Youngeun Chu, KiYoung Lee, Eung Il Kim

**Affiliations:** School of Business, Yonsei University, Seoul 03722, Korea; chuxx086@umn.edu (Y.C.); kei0319@yonsei.ac.kr (E.I.K.)

**Keywords:** sleep, employee health, work engagement, workplace aggression

## Abstract

Recent studies have shown that workplace victimization is negatively related to work engagement. The explanations for the underlying mechanisms, however, are still in a nascent stage. Drawing on the limited resource theory of self-regulation and research on workplace aggression and sleep, we develop and test an integrated model, which explains that victimized employees may have impaired sleep quality and thus have less energy and be less likely to be engaged in their work. The results of logistic regression and structural equation modeling analyses of large-scale survey data collected from 90,272 employees across the years 2010, 2011, 2014, and 2017, indicate that workplace victimization is negatively related to sleep quality and subsequent workplace engagement, even controlling for alternative explanations—job insecurity and basic psychological needs for competence, autonomy, and relatedness. Our findings advance our knowledge on the detrimental consequences of workplace victimization and suggest that, while unmet basic psychological needs matter, impaired sleep quality is one reason why victimized employees find it difficult to engage at work.

## 1. Introduction

Workplaces often feature negative interpersonal interactions [1,2,3,4,5,6], such as coworker undermining [7,8], supervisor abuse [9,10], subordinate defiance [11,12], and customer mistreatment [13,14]. Negative interpersonal experiences occur less frequently than positive interactions but have larger impacts on employee well-being, attitudes, and functioning (cf. [15]). As such, researchers have long observed the consequences of what is broadly termed workplace victimization [16,17,18].

Researchers vary in their conceptualization of negative interactions at work and use different labels (see [18]). Aquino et al. [16] (1999, p. 260) defined victimization as “an individual’s perception of having been exposed, either momentarily or repeatedly, to the aggressive acts of one or more other persons.” The definition is broad in that victimization can occur “when an employee’s well-being is harmed by an act of aggression” ([18]). In our study, we use this definition and consider victimized employees as those who are targets of aggression, whether through bullying, physical violence, or sexual harassment. Predictably, workplace victimization is related to damaged well-being and attitudes (for reviews, see [18,19,20,21]), psychological distress [22,23], sickness [24,25,26], and job dissatisfaction [27,28]. Victimized employees often retaliate by displaying deviant behaviors towards their coworkers, supervisors, and organizations [7,29,30,31,32,33,34].

Workplace victimization can also reduce *work engagement* (e.g., [35,36,37,38,39,40,41,42,43,44,45,46], which is defined as a broad motivational state that drives employees to devote their full supply of energy to their work [47]. Engagement is a critical antecedent to performance [48,49], more so than other known predictors such as job involvement, job satisfaction, and intrinsic motivation [50]. Despite some evidence showing that victimization is linked to a critical motivational state of work engagement, this area has been sparsely researched [36], so we know little about why victimization impairs work engagement.

In this study, we aimed to study the theoretical mechanisms involved in the relationship between victimization and work engagement. We drew upon the limited resource theory of self-regulation [51,52] as a key underlying process that explains why victimization impairs work engagement. This theory posits that individuals have limited resources to devote to self-regulation efforts, and depleted resources must be properly restored before self-regulation is possible. For example, immediately after devoting many hours of emotional labor to a customer, employees might find it difficult to concentrate on work tasks without checking emails. As a stressful experience, victimization causes constant and intrusive ruminations [7,53] that interrupt resource recovery through sleep and deplete the energy available for work engagement. Sleep quality, defined as “difficulty of falling asleep, staying asleep, and the number of awakenings experienced in the night” [54], is a key indicator of self-regulation restoration [54,55]. In this study, we theorize and test whether sleep quality is an important mediator that can help to explain why victimization impairs work engagement.

Our study makes three major contributions to the literature. First, we extend the list of negative outcomes associated with workplace victimization (see [18] for a review) by examining how workplace victimization is linked to employees’ sleep. Compared to theoretical discussion on workplace victimization and sleep, empirical evidence is lacking (for exceptions, see [56,57,58]). For our study, we use data from a large-scale survey of 90,272 employees collected over 4 years. Our aim is to provide robust evidence that workplace victimization is linked to impaired sleep quality, so that this can be added to other known consequences described in the literature, such as job dissatisfaction, impaired well-being, and destructive retaliation.

Second, we aim to contribute to the growing effort to incorporate sleep research into management literature (for reviews see [54,59]). Sleep is one of the most important recovery activities and is essential for human functioning [60,61], particularly for work activities that usually require self-regulation (for a review see [62]). As such, sleep influences numerous employee states and work behaviors [54]. Sleep research shows that stressful experiences interfere with the recovery processes that should occur during sleep [63,64]. We examine whether the impaired sleep quality associated with workplace victimization has downstream consequences such as a low level of work engagement.

Third, and most importantly, our study aims to examine the processes underlying the link between victimization and work engagement. Combining literature on self-regulation, workplace aggression, and sleep, we test a mediation model to investigate whether impaired sleep quality explains why victimized employees become less engaged at work. In particular, we take a comprehensive approach and include previously examined mediators of job insecurity [43] and basic psychological needs [38] as alternative mechanisms within the same model, and evaluate the relative levels of practical importance of competing theories.

In the following text, we first outline the theoretical background of our study more fully and derive hypotheses. We then report the results of hypothesis testing based on data from a large survey of 90,272 Korean employees collected over four years. For the first three years of data (years 2010, 2011, 2014), we focus on the relationship between victimization and sleep quality. Using data from the last year (year 2017), we test the mediating role of sleep quality on the relationship between victimization and work engagement while controlling for alternative mediators. We conclude our study with a discussion on the study’s findings.

## 2. Theoretical Background and Hypotheses

### 2.1. Victimization and Sleep Quality

Employees often become targets of interpersonal aggression from supervisors, coworkers, subordinates, and customers [1,11,14,28]. Inconsiderate and disrespectful treatment violates social interaction norms [65]. Victimized employees then become preoccupied with trying to interpret the meaning behind the treatment [7,53]. For instance, they may obsess about why they were treated so rudely, whether they were somehow responsible, and how they should act if it happens again. These cognitive rumination processes are often intrusive, difficult to control [66], and persistent, even after work hours [67].

Sleep quality is impaired when individuals have difficulty falling asleep and wake frequently [60]. When employees have stressful interpersonal experiences at work, they find it difficult to cognitively unwind and stop worrying [56,58,68]. As victimized employees often recall abusive episodes and cognitively prepare themselves for future interactions, they become physiologically activated and have difficulty falling and staying asleep, waking several times during the night [54,68,69]. Although few studies have explicitly considered the link between workplace victimization and sleep, abusive supervision [57] and ostracization at work [56,58] have been shown to undermine sleep quality. Thus, we argue that impaired sleep quality is a detrimental outcome of negative interpersonal treatment at work, particularly workplace victimization, which suggests the following hypothesis:

**Hypothesis** **1.** *Workplace victimization is negatively related to sleep quality.*

### 2.2. Sleep Quality and Work Engagement

Sleep research has shown that sleep allows recovery from daily experiences and rebuilds an individual’s energy and resources for the next day [70,71]. Impaired sleep quality has particularly crucial effects on many human states and functions [72], such as their daily mood [73], health [74], likelihood of experiencing an accident or injury [75,76,77], and performance (e.g., [57,78,79]).

We draw on the limited resource theory of self-regulation [51,52], which argues that people have a limited capacity to override natural responses in daily efforts to meet social and other standards [52,80,81]. The tenet of the theory is that self-regulation is drawn from a limited amount of resources. Unless resources are restored, people find it difficult to self-regulate in subsequent activities. Workplaces require self-regulatory efforts during various activities such as concentrating on tasks without being distracted and using socially appropriate friendly behavior toward customers. When sleep fails to properly restore self-regulatory resources, employees cannot summon the self-regulation needed to concentrate on tasks [79,82], avoid procrastination [83], comply with safety regulations [76,84], and treat others respectfully [72].

Aligned with this reasoning and evidence, we predict that poor sleep quality is negatively associated with the broader motivational state of work engagement, which requires physical, cognitive, and emotional resources [48,50]. Therefore, when employees lack the sleep needed to recover resources, they lack reserves for concentrating and devoting energy to their jobs. Surprisingly, only a few studies have examined the relationship between sleep quality and work engagement (e.g., [70,83]). We hypothesize the following:

**Hypothesis** **2.** *Sleep quality is positively related to work engagement.*

### 2.3. The Mediating Role of Sleep Quality on the Relationship between Victimization and Work Engagement

We hypothesize that victimization reduces sleep quality (Hypothesis 1), which then reduces work engagement (Hypothesis 2). Combining our hypotheses, we expect that impaired sleep quality partially explains why victimization is negatively related to work engagement.

Many studies that have examined links between victimization and work engagement have provided unclear underlying theoretical mechanisms (e.g., [45]). Some have used general stress-based frameworks (e.g., [36,37]), positing that victimization inflicts stress and strain. Others have explicitly tested underlying mechanisms (e.g., [35,38,43,44]) to show, for instance, that victimized employees are disengaged at work because they perceive their workplace to be unsafe [35] or consider that an implicit breach of psychological contracts regarding respectful treatment has occurred [44]. Theorizing victimization threatens self-worth and social inclusion, leading to fear of a potential job loss, a study also showed that job insecurity mediates the relationship between victimization and work engagement [43]. Based on self-determination theory (SDT) [85], unmet basic psychological needs were used to explain why victimization impairs work engagement. Specifically, victimized employees are deprived of a sense of ownership at work (need for autonomy), are prevented from achieving goals (need for competence), and feel isolated at work (need for relatedness) [38,86].

These studies increased our understanding of why victimized employees may lack work engagement, but we propose that impaired sleep quality is an additional explanatory mechanism. The lack of studies examining sleep as a mediator is surprising, considering that the theoretical rationale can be derived by connecting workplace aggression, self-regulation, and sleep literature. Because victimized employees are likely to have disturbed sleep and sleep is a recovery activity that has important implications for everyday functioning, we predict that sleep quality partially mediates the relationship between victimization and work engagement. Given the theoretical distinctiveness and usefulness of the limited resource theory for self-regulation for explaining numerous employee outcomes (for reviews see [87,88]) and the broad effects of sleep quality on employee functioning (for a review see [54]), we hypothesize that the proposed indirect relationship between victimization and work engagement via sleep quality holds, even after controlling for alternative mechanisms:

**Hypothesis** **3.** *Victimization is negatively related to work engagement via sleep quality after controlling for job insecurity and basic psychological needs for competence, autonomy, and relatedness.*

## 3. Method

We used the Korean Working Conditions Survey (KWCS), a public database collected by the Occupational Safety and Health Research Institute (OSHRI) in Korea [89]. The KWCS comprises non-panel, cross-sectional survey datasets for five-year periods and primarily includes survey questions on workplace safety, health, and stress. Using a multistage random sampling approach, datasets for four years—2010, 2011, 2014, and 2017—were used to reflect a nationally representative sample. Participants were aged 15 and over and worked in various occupations. The sample included self-employed, business owners, salaried workers, and unpaid family employees. The sample used for analysis included paid workers to obtain information about the effects of victimization experiences from various perpetrators such as supervisors, coworkers, subordinates, and clients.

The 2010, 2011, and 2014 datasets included measures of workplace victimization and sleep quality and were thus used to test the victimization–sleep relationship (Hypothesis 1). Because the datasets were not panel-based, we analyzed the data for each year separately to test Hypothesis 1. The 2017 dataset included data on workplace victimization, sleep quality, work engagement, and variables representing basic psychological needs and job insecurity, the alternative mechanisms. Thus, in addition to Hypothesis 1, the 2017 dataset allowed us to test Hypothesis 2, the sleep quality–work engagement link, and Hypothesis 3, whether sleep quality is a mediator after controlling for alternative mechanisms.

**Figure 1:**
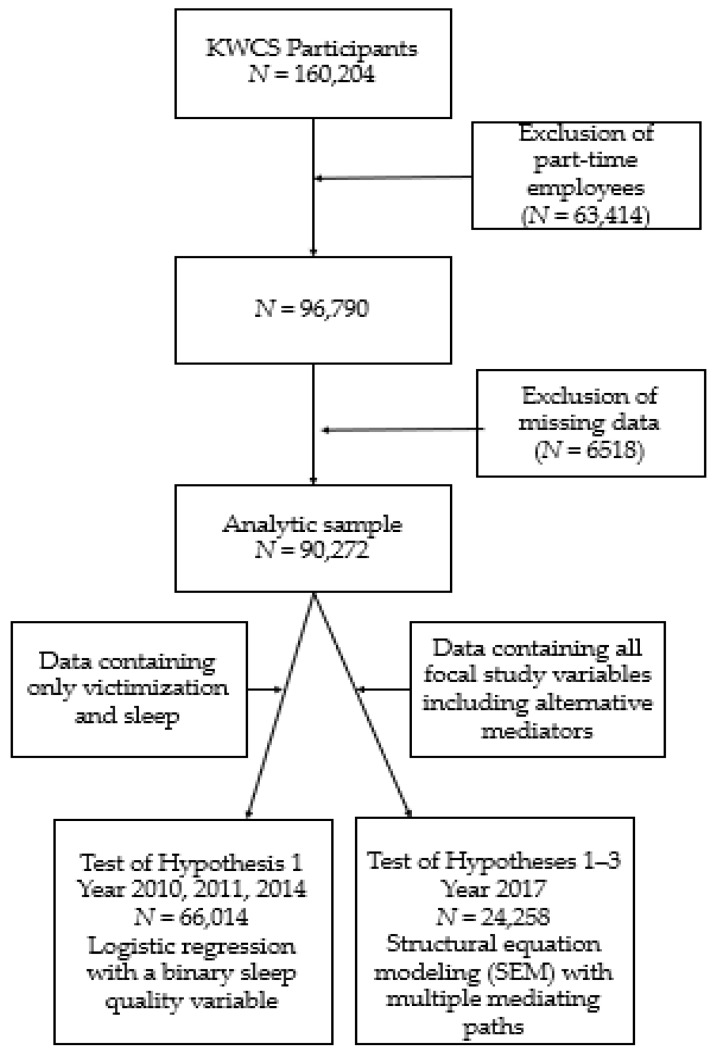
A flow chart explaining the process of selecting the final sample and method used for the analysis.

Of the 160,204 participants sampled across four years, we excluded part-time employees (N = 63,414) and restricted the sample to 96,790 full-time workers: 47.30% were women; the average age was 44.71 years (*SD* = 12.98); 87.4% had a high school or higher degree; 1.3% were uneducated; 4.1% had only completed elementary school; 7% had completed only up to middle school; 37.7% only had a high school degree; 16.6% had a technical school certificate; 30.7% had a bachelor’s degree; and 2.3% had a master’s degree or above. Participants were full-time employees in positions such as sales (19.6%), white-collar office jobs (12.9%), and service positions (16.1%). Excluding observations with missing values on study variables, the final sample for analysis was reduced to 90,272 (6220 for 2010; 29,711 for 2011; 30,083 for 2014; 24,258 for 2017). The flow chart in Figure 1 explains the process of selecting the analytic sample.

## 4. Measures

The KWCS used survey items translated from the European Working Conditions Survey (EWCS). Following the guidelines set by the EWCS for translation into different languages and Brislin’s (1980) [90] procedure, bilingual assistants first translated the original English items into Korean. Other bilingual assistants then back-translated the Korean items into English. They used iterative processes to ensure equal meanings were present. Finally, experts in safety, health care, and surveys examined and discussed the appropriateness of the final survey items for a Korean audience.

Victimization was measured with three items—physical violence, sexual harassment, and bullying/harassment. Each item was preceded by the question, “Over the past 12 months, during the course of your work have you been subjected to any of the following?” For our analysis, responses were coded *no* = 0 and *yes* = 1 for each of three types of victimization. For those who responded “yes,” a follow-up question asked whether victimizers were (1) coworkers, supervisors, or subordinates or (2) clients. We treated victimization as an index score that comprehensively captures types of victimization and perpetrators. Therefore, we summed responses (0/1) to create a composite index score of victimization experience, potentially from 0 to 3.

For the first three years of data—2010, 2011, and 2014—sleep quality was measured with a single item: “Over the past 12 months, did you have any of the following health problems (sleep problems, insomnia)?” For our analysis, responses were coded *yes* = 0, *no* = 1. A score of 1 indicated better sleep quality. For 2017, participants were asked the question, “Over the past 12 months, how often did you have any of the following sleep-related problems?” Items were “difficulty falling asleep”; “waking repeatedly”; and “waking feeling exhausted and fatigued” (from 1 = daily to 5 = never) (α = 0.87). The three items for the year 2017 were comparable to items widely used in a sleep questionnaire developed by Jenkins and colleagues (1988) [91]. The items are: “Had trouble falling asleep”; “Had trouble staying asleep (including waking up too early)”; “Woke up several times during the night”; and “Woke up after the usual amount of sleep feeling tired and worn out” [92] (see also, [69,92]).

Work engagement was measured in the 2017 dataset. Participants responded to the statements, “At my work I feel full of energy”; “I am enthusiastic about my job”; “Time flies when I am working”; and “I think I am good at work” (1 = never to 5 = always) (α = 0.77). The four items corresponded to vigor, dedication, and absorption components on the work engagement scale [93,94].

Using the 2017 data, we tested whether sleep quality, our focal mediator, held after controlling for job insecurity and basic psychological needs for autonomy, relatedness, and competence as alternative mediators. Job insecurity was measured with three items [95], for example, “I might lose my job in the next 6 months”, which were answered on a five-point Likert-type scale (1 = strongly agree to 5 = strongly disagree) and reverse coded. Autonomy was measured with four items, for example, “You can influence decisions that are important for your work” (α = 0.82). Relatedness was measured with three items, for example, “Your colleagues help and support you” (α = 0.66). Competence was measured with three items, for example, “I receive the recognition I deserve for my work” (α = 0.69). The three variables were answered on a five-point Likert-type scale (1 = strongly agree to 5 = strongly disagree). We reverse-coded original scores so that higher scores represented greater needs fulfillment.

## 5. Analysis Strategy

To test our hypotheses, we ran logistic regression and structural equation modeling (SEM) in STATA 16.0 [96]. First, for the years 2010, 2011, and 2014, sleep quality was measured on a binary scale. When the dependent variable is a dichotomous variable, logistic regression is an appropriate analysis method [97]. Second, the year 2017 data included all focal study variables, including alternative mediators. When multiple mediators are present in the same model, SEM is an appropriate method to test indirect effects as it considers multiple paths simultaneously [98]. For the significance testing of indirect effects, we used a bootstrapping method involving the construction of confidence intervals based on 5000 samples [99].

## 6. Results

Table 1 shows the descriptive statistics and correlations among the study variables for the years 2011, 2011, and 2014. Victimization was found to be negatively correlated with sleep quality in each year (year 2010: *r* = −0.06, *p* < 0.01; year 2011: *r* = −0.04, *p* < 0.01; year 2014: *r* = −0.07, *p* < 0.01).

Table 2 shows the results of the logistic regression analysis used to test Hypothesis 1. Victimization was found to be negatively related to sleep quality for each year (year 2010: B = −0.13, *p* < 0.01. odds ratio = 0.88; year 2011: B = −0.11, *p* < 0.01. odds ratio = 0.89; year 2014: B = −0.15, *p* < 0.01. odds ratio = 0.87). In terms of the odds ratio [100,101], a one-unit increase in victimization was shown to reduce the sleep quality by about 0.88 (year 2010), 0.89 (year 2011), and 0.87 times (year 2014), respectively. Therefore, Hypothesis 1 was supported.

Table 3 shows the descriptive statistics and correlations among the study variables for the 2017 data, including the focal variables used for testing Hypotheses 1–3. As expected, victimization was found to be negatively correlated with sleep quality (*r* = −0.05, *p* < 0.01) and work engagement (*r* = −0.02, *p* < 0.01). Furthermore, sleep quality was found to be positively correlated with work engagement (*r* = 0.12, *p* < 0.01). As expected, the three basic psychological needs also showed negative correlations with victimization (autonomy: *r* = −0.03, *p* < 0.01; competence: *r* = −0.04, *p* < 0.01; relatedness: *r* = −0.03, *p* < 0.01) and positive correlations with work engagement (autonomy: *r* = 0.29, *p* < 0.01; competence: *r* = 0.52, *p* < 0.01; relatedness: *r* = 0.50, *p* < 0.01). Job insecurity was not found to be significantly correlated with victimization (*r* = −0.00, n.s.) but it was negatively correlated with work engagement (*r* = −0.09, *p* < 0.01).

Before we used SEM to test Hypotheses 1–3 using data from the year 2017, we assessed the fit of the measurement model [102]. Because victimization was operationalized as an index score, we modeled victimization as one observed variable. All other variables were modeled as latent variables with items as indicators. Table 4 reports the fit indices for our focal and alternative models. Our study model fitted the data well (χ^2^ = 10,930.39, *df* = 151, comparative fit index (CFI) = 0.94, Tucker–Lewis index (TLI) = 0.92, root mean square error of approximation (RMSEA) = 0.05, standardized root mean square residual (SRMR) = 0.05) [103], significantly better than other alternative models. The three alternative models were (1) autonomy, competence, relatedness, and sleep combined into a single factor (alternative model A; χ^2^ = 95,713.95, *df* = 166, CFI = 0.43, TLI = 0.35, RMSEA = 0.15, SRMR = 0.20, Δχ^2^ = 84,783.55); (2) victimization, sleep quality, and work engagement combined into a single factor (alternative model B; χ^2^ = 44,732.564, *df* = 161, CFI = 0.72, TLI = 0.67, RMSEA = 0.11, SRMR = 0.15, Δχ^2^ = 36,802.17); and (3) autonomy, competence, relatedness, and job security combined into a single factor (alternative model C; χ^2^ = 11,254.47, *df* = 156, CFI = 0.93, TLI = 0.92, RMSEA = 0.05, SRMR = 0.05, Δχ^2^ = 324.08).

**Figure 2:**
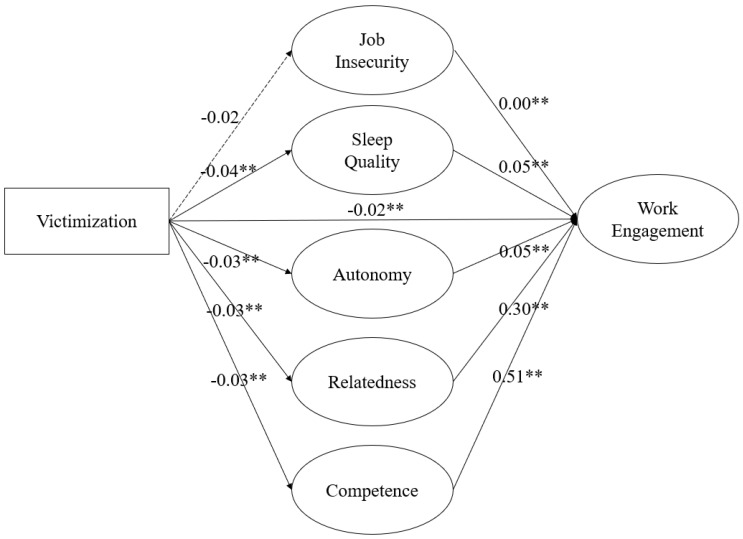
Results of structural equation modeling (year 2017). *Notes: N* = 24,258, *p* < 0.01 **. Victimization was modeled using a composite index and is represented by a rectangle. All other variables are represented by circles and were modeled as latent constructs. The dotted lined indicates a non-significant relationship.

After confirming that the model has a good fit for the measurement model, we used SEM to test our hypotheses using a multiple mediation model. As Figure 2 shows, victimization was found to be negatively related to sleep quality (*β*= −0.04, *p* < 0.01), thus supporting Hypothesis 1. Supporting Hypothesis 2, sleep quality was found to be positively related to work engagement (*β* = 0.05, *p* < 0.01).

Hypothesis 3 predicts that sleep quality mediates the relationship between victimization and work engagement after controlling for job insecurity and basic psychological needs as alternative mediators. We calculated the product of coefficients for indirect effect using the path coefficients from victimization to sleep quality (Hypothesis 1) and from sleep quality to work engagement (Hypothesis 2). Results based on 5000 bootstrapped samples and constructed confidence intervals [99] showed a negative and statistically significant indirect effect of victimization on work engagement via sleep quality (indirect effect = −0.002), 95%CI [−0.003, −0.001]). Therefore, Hypothesis 3 was supported.

With regard to results of other indirect effects (Figure 2 and Table 5), victimization was not found to be significantly related to job insecurity (*β=* −0.02, n.s.), although job insecurity was shown to be positively related to work engagement (*β* = 0.00, *p* < 0.01). The indirect effect via job insecurity was also not shown to be significant (indirect effect = 0.00, n.s.). In terms of basic psychological needs, victimization was found to be negatively related to the need for autonomy (*β* = −0.03, *p* < 0.01), need for relatedness (*β* = −0.03, *p* < 0.01), and need for competence (*β* = −0.03, *p* < 0.01). All three basic psychological needs showed significant indirect effects (for autonomy, indirect effect = −0.001, 95%CI [−0.002, −0.001]; for relatedness, indirect effect = −0.008, 95%CI [−0.011, −0.005]; for competence, indirect effect = −0.011, 95%CI [−0.015, −0.007]).

## 7. Discussion

In this study, we developed and tested a model to explain why victimized employees tend to have impaired work engagement. We first established the link between victimization and sleep quality using data collected in a large-scale survey covering the years 2010, 2011, and 2014. We then used data from 2017 to show that low sleep quality partially explains why workplace victimization impairs engagement at work, and the relationship held after we controlled for job insecurity and basic psychological needs for competence, autonomy, and relatedness as alternative mechanisms.

Our work explicates underlying theoretical processes for the victimization–work engagement link. Despite growing interest in how victimization reduces work engagement, theoretical advances have been slow because studies have used a general stress framework [36] or examined mediators in isolation [35,38]. We integrated theories and research on workplace aggression, self-regulation, and sleep to propose sleep quality as a mediator and then test alternative mediators of job insecurity and basic psychological needs. To our knowledge, no previous studies have examined sleep quality as a mediator in the workplace victimization–engagement relationship comprehensively with other mediators. By combining multiple mediators in the same model, our approach revealed the theoretical and practical value of competing theories [104]. Our results show that the indirect effect via sleep on the victimization-work engagement link accounts for 7.80% of the total indirect effect and we found this effect above and beyond the three basic psychological needs and job insecurity. It is also worth comparing the results of our study to those from previous models described in the literature. Based on self-determination theory, Goodboy et al. (2020) [38] and Trépanier et al. (2013) [86] studied the effects of the three basic psychological needs [85] as mediators on the relationship between victimization and work engagement. While Trépanier et al. (2013) [86] found that all three of the basic psychological needs significantly mediate the relationship, Goodboy et al. (2020) [38] showed that the need for competence does not mediate the relationship. The results of our study show that all three basic psychological needs are important mediators, with the needs for relatedness and competence, in particular, having relatively larger indirect effects, explaining 35.78% (via relatedness) and 50.46% (via competence) of the total indirect effect in the model. Thus, although the focus of our study was the role of sleep quality, our results show that basic psychological needs might explain a larger portion of the phenomenon. We encourage future studies to confirm this and to include basic psychological needs in studies of new mediators. In addition, Park and Ono (2017) [43] found job insecurity to be a significant mediator on the victimization-work engagement link. In our study, job insecurity was not found to have a significant indirect effect. However, rather than undervaluing job insecurity as a mediator, we must recognize that it might play a more complex role. For instance, the underlying rationale for the mediation role of job insecurity on the relationship between victimization and work engagement was that victimization threatens self-worthiness and social inclusion, both of which are closely related to needs for competence and relatedness under the SDT framework [43]. We explored a potential serial mediation in which victimization lowers the need for competence and relatedness (as well as sleep quality and need for autonomy), which, in turn, increases the perception of job insecurity and therefore influences work engagement. Although each path of the model was found to be significant and in the predicted direction, the model showed a worse fit (χ^2^ = 32,016.07, *df* = 163, CFI = 0.81, TLI = 0.78, RMSEA = 0.09, SRMR = 0.17). Given the nascent stage of this research, we encourage future studies to examine the role of job insecurity using a more refined model and research design.

Our work broadens the workplace victimization literature by introducing sleep quality as a home outcome, which supports previous work showing that victimized employees carry victimization consequences into their home lives. It is already known that mistreatment at work impacts home life through displaced aggression [105,106] or withdrawal behaviors toward family members [107]. Our study goes further to show that victimized employees carry consequences to bed. Thus, mistreatment at work depletes resources by impairing sleep quality. The effects cycle back to the workplace by lowering work engagement.

### 7.1. Limitations and Future Directions

We acknowledge that our study has limitations. Firstly, cross-sectional survey data yield findings that cannot rule out other causal orderings. An alternative model may suggest that employees who are not engaged at work are likely to be seen as less competent and sociable and, thus, are victimized. We tested alternative orderings by examining whether sleep quality and needs for competence and relatedness mediate the relationship between work engagement and victimization. Though each path of the model was significant and in the predicted direction, the model a showed worse fit (χ^2^ = 23,386.44, *df* = 140, CFI = 0.84, TLI = 0.81, RMSEA = 0.08, SRMR = 0.15). It is also possible that employees with poor sleep quality are more susceptible to victimization as they have impaired emotional regulation and experience a negative mood and burnout, and victimization subsequently reduces work engagement. Although this alternative ordering of “sleep quality → victimization → work engagement” showed a good fit (χ^2^ = 9854.88, *df* = 192, CFI = 0.93, TLI = 0.92, RMSEA = 0.05, SRMR = 0.05), victimization was not found to be significantly related to work engagement (*β* = 0.01, n.s.). Future studies could use a longitudinal design to increase the level of confidence in causality and allow more complex relationships among the variables to be examined.

Secondly, it might be argued that our significant results are due to the large sample size and associated power. We believe, however, that the results are meaningful because our dataset is a nationally representative sample. Importantly, our models were grounded in theories with the focal mechanism being compared against multiple alternative theories.

Thirdly, we measured sleep quality but not quantity. We theorized that victimization evokes intrusive thoughts that wake victimized employees several times during the night. Frequent waking would interfere with sleep quality, but interrupted sleep would also be related to sleep quantity. Sleep quality and quantity are distinct concepts, but both limit the restoration of resources and are thus expected to have similar additive effects [54]. Sleep quantity was untested as a mediator because the data failed to provide that information. Future studies should examine both the quantity and quality of sleep to provide a more comprehensive understanding of the effect of sleep on the link between victimization and work engagement.

Finally, by including alternative mechanisms, our study is more comprehensive than previous studies that examined the victimization–work engagement phenomena, but other possibilities should be considered. For instance, psychological contract violation [44] and perceptions of unsafety [35] may partially explain the link between victimization and work engagement. Our dataset, however, allowed us to control for job insecurity [43] and for three basic psychological needs, factors that are highly relevant for explaining work motivation [108,109] and have been studied in terms of their roles as mediators in victims’ work engagement (e.g., [38,86]). We believe that our study provides a more complete picture of victimization–work engagement phenomena rather than building isolated explanations [110]. Future studies could continue our effort by considering plausible alternative mechanisms.

### 7.2. Practical Implications

Our work suggests that victimization has far-reaching detrimental consequences beyond being an unpleasant momentary experience, as it may inhibit the restorative sleep employees need to have energy to devote to their work. Therefore, we advise organizations to provide training and education to prevent victimization in the workplace. For instance, CREW (Civility, Respect, Engagement in the Workforce) intervention [111] is recommended to instill respect, care, and awareness of consequences. In addition, organizations are advised to provide comprehensive support to help victimized employees to learn how to recover from mistreatment as well as how to acquire the sleep they need to focus on their work. Managers should be aware that proactive approaches are needed because suffering may be invisible. For instance, organizations could periodically offer regular counseling, including sleep therapy.

## 8. Conclusions

In conclusion, this study shows that impaired sleep quality explains why victimized employees are less engaged at work. The mediation effect held even when we accounted for job insecurity and the three basic psychological needs, which were previously examined in the literature. Thus, we suggest that sleep quality is a mediator that deserves attention as it could enhance our understanding of the victimization–work engagement link.

## Figures and Tables

**Table 1:** Descriptive statistics and correlations for the years 2010, 2011, and 2014.

Variable	Mean	Standardized Deviation	Correlation
Victimization_2010_	0.01	0.10	
Sleep Quality_2010_	0.97	0.17	−0.06 **
Victimization_2011_	0.02	0.16	
Sleep Quality_2011_	0.98	0.15	−0.04 **
Victimization_2014_	0.01	0.11	
Sleep Quality_2014_	0.97	0.17	−0.07 **

*Note. p* < 0.01 **. *N*_2010_ = 6220, *N*_2011_ = 29,711, *N*_2014_ = 30,083.

**Table 2:** Results of logistic regression analysis with victimization and sleep quality.

Variable	B (SE)	Wald	Sig.	95% C.I. for Odds Ratio		
				Lower	Odds Ratio	Upper
Victimization_2010_	−0.13(0.04)	13.97	0.00	0.82	0.88	0.94
Victimization_2011_	−0.11(0.02)	34.52	0.00	0.86	0.89	0.93
Victimization_2014_	−0.15(0.02)	85.10	0.00	0.84	0.87	0.89

*Note. N*_2010_ = 6220, *N*_2011_ = 29,711, *N*_2014_ = 30,083.

**Table 3:** Descriptive statistics and correlations for the year 2017.

Variable	Mean	S.D.	1	2	3	4	5	6	7
1. Victimization	0.01	0.11							
2. Sleep Quality	4.46	0.72	−0.05 **	(0.87)					
3. Work Engagement	3.54	0.57	−0.02 **	0.12 **	(0.77)				
4. Job insecurity	1.84	1.04	−0.00	−0.18 **	−0.09 **				
5. Autonomy	3.16	0.79	−0.03 **	−0.05 **	0.29 **	0.00	(0.82)		
6. Competence	3.70	0.51	−0.04 **	0.12 **	0.52 **	−0.07 **	0.35 **	(0.66)	
7. Relatedness	3.43	0.61	−0.03 **	0.13 **	0.50 **	−0.13 **	0.40 **	0.54 **	(0.69)

*Note. N* = 24,258, *p* < 0.01 **. Numbers on diagonal represent coefficient alphas.

**Table 4:** Summary of model fit indexes.

	χ^2^ (*df*)	Δχ^2^	CFI	TLI	RMSEA	SRMR
Full model	10,930.39 (151)		0.94	0.92	0.05	0.05
Alternative Model A ^a^	95,713.95 (166)	84,783.55 **	0.43	0.35	0.15	0.20
Alternative Model B ^b^	47,732.56 (161)	36,802.17 **	0.72	0.67	0.11	0.15
Alternative Model C ^c^	11,254.47 (156)	324.08 **	0.93	0.92	0.05	0.05

*Note. N* = 24,258, *p* < 0.01 **, ^a^ autonomy, competence, relatedness, and sleep combined into a single factor, compared to the full measurement model; ^b^ victimization, sleep quality, and work engagement combined into a single factor, compared to the full measurement model; ^c^ autonomy, competence, relatedness and job insecurity combined into a single factor, compared to the full measurement model.

**Table 5:** Indirect effects of victimization on work engagement via sleep quality, job insecurity, and the three basic psychological needs (year 2017).

	Bootstrapping	Bias-Corrected 95% CI		Proportion of Indirect Effect (%)
Paths	Estimate	Lower	Upper	
Victimization → Sleep quality → Work engagement	−0.002	−0.003	−0.001	7.80
Victimization → Job insecurity → Work engagement	0.000	0.000	0.000	0.00
Victimization → Autonomy → Work engagement	−0.001	−0.002	−0.001	5.96
Victimization → Relatedness → Work engagement	−0.008	−0.011	−0.005	35.78
Victimization → Competence → Work engagement	−0.011	−0.015	−0.007	50.46
Total indirect effect	−0.022	−0.029	−0.015	100

## Data Availability

Publicly available datasets were analyzed in this study. The datasets are available here: https://www.kosha.or.kr/eoshri/resources/KWCSDownload.do (accessed on 7 August 2021).
